# Supplementary material for: Kinetics of the xanthophyll cycle and its role in photoprotective memory and response
Source: Nat Commun. 2023 Oct 19;14:6621. doi: 10.1038/s41467-023-42281-8 (PMC10587229; doi:10.1038/s41467-023-42281-8)
Supplement: Supplementary file 1 — Supplementary Information [file 41467_2023_42281_MOESM1_ESM.pdf]

# Supplementary Information to: "Kinetics of the Xanthophyll Cycle and its Role in the Photoprotective Memory and Response"

Audrey Short,[1,2,3,a)] Thomas P. Fay,[4,a)] Thien Crisanto,[2,5,6] Ratul Mangal,[4] Krishna K. Niyogi,[2,5,6] David T. Limmer,[1,3,4,7,8] and Graham R. Fleming[1,2,3,4,b)]
[1)] *Graduate Group in Biophysics, University of California, Berkeley, CA 94720 USA*
[2)] *Molecular Biophysics and Integrated Bioimaging Division Lawrence Berkeley National Laboratory, Berkeley, CA 94720 USA*
[3)] *Kavli Energy Nanoscience Institute, Berkeley, CA 94720 USA*
[4)] *Department of Chemistry, University of California Berkeley, CA 94720 USA*
[5)] *Department of Plant and Microbial Biology, University of California, Berkeley, CA 94720 USA*
[6)] *Howard Hughes Medical Institute, University of California, Berkeley, CA 94720 USA*
[7)] *Chemical Science Division Lawrence Berkeley National Laboratory, Berkeley, CA 94720 USA*
[8)] *Material Science Division Lawrence Berkeley National Laboratory, Berkeley, CA 94720 USA*

## CONTENTS

**I. Further Model details** 1

**II. Reduced model for HPLC data** 3

**III. Model parameters** 3

**IV. Mechanism of qZ** 4

**V. Estimating quenching rates** 5

**VI. Raw HPLC data** 5

**VII. NPQ recovery in 5 HL-*T* D-5 HL sequences** 6

**References** 6

## I. FURTHER MODEL DETAILS

The kinetic scheme for our VAZ cycle based model of non-photochemcial quenching in *Nanno* is given explicitly here

$$\mathrm{P} + \mathrm{V} \underset{k_{\mathrm{PV,b}}}{\overset{k_{\mathrm{PV,f}}}{\rightleftharpoons}} \mathrm{PV} \underset{k_{\mathrm{QV,b}}^{\mathrm{light/dark}}}{\overset{k_{\mathrm{QV,f}}^{\mathrm{light/dark}}}{\rightleftharpoons}} \mathrm{QV} \tag{1}$$

$$\mathrm{P} + \mathrm{A} \underset{k_{\mathrm{PA,b}}}{\overset{k_{\mathrm{PA,f}}}{\rightleftharpoons}} \mathrm{PV} \underset{k_{\mathrm{QA,b}}^{\mathrm{light/dark}}}{\overset{k_{\mathrm{QA,f}}^{\mathrm{light/dark}}}{\rightleftharpoons}} \mathrm{QA} \tag{2}$$

$$\mathrm{P} + \mathrm{Z} \underset{k_{\mathrm{PZ,b}}}{\overset{k_{\mathrm{PZ,f}}}{\rightleftharpoons}} \mathrm{PV} \underset{k_{\mathrm{QZ,b}}^{\mathrm{light/dark}}}{\overset{k_{\mathrm{QZ,f}}^{\mathrm{light/dark}}}{\rightleftharpoons}} \mathrm{QZ} \tag{3}$$

$$\mathrm{V} + \mathrm{VDEa} \underset{k_{\mathrm{A}\to\mathrm{V}}}{\overset{k_{\mathrm{V}\to\mathrm{A}}}{\rightleftharpoons}} \mathrm{A} + \mathrm{VDEa} \underset{k_{\mathrm{Z}\to\mathrm{A}}}{\overset{k_{\mathrm{A}\to\mathrm{Z}}}{\rightleftharpoons}} \mathrm{Z} + \mathrm{VDEa} \tag{4}$$

$$\mathrm{VDEi} \underset{k_{\mathrm{VDE,b}}^{\mathrm{light/dark}}}{\overset{k_{\mathrm{VDE,f}}^{\mathrm{light/dark}}}{\rightleftharpoons}} \mathrm{VDEa}. \tag{5}$$

---

[a)] These authors contributed equally.
[b)] Electronic mail: grfleming@lbl.gov

Each step is treated as an elementary rate process in constructing kinetic equations for the chemical species. The full set of kinetic equations is therefore

$$\frac{\mathrm{d}}{\mathrm{d}t}[\mathrm{X}] = -k_{\mathrm{PX,F,eff}}[\mathrm{X}][\mathrm{P}] + k_{\mathrm{PX,b}}[\mathrm{PX}], \quad \text{for X = V,A,Z} \tag{6}$$

$$\frac{\mathrm{d}}{\mathrm{d}t}[\mathrm{PX}] = k_{\mathrm{PX,F,eff}}[\mathrm{X}][\mathrm{P}] - k_{\mathrm{PX,b}}[\mathrm{PX}] - k^{\mathrm{light/dark}}_{\mathrm{QX,F,eff}}[\mathrm{PX}] + k^{\mathrm{light/dark}}_{\mathrm{QX,b}}[\mathrm{QX}], \quad \text{for X = V,A,Z} \tag{7}$$

$$\frac{\mathrm{d}}{\mathrm{d}t}[\mathrm{QX}] = k^{\mathrm{light/dark}}_{\mathrm{QX,F,eff}}[\mathrm{PX}] - k^{\mathrm{light/dark}}_{\mathrm{QX,b}}[\mathrm{QX}], \quad \text{for X = V,A,Z} \tag{8}$$

$$\frac{\mathrm{d}}{\mathrm{d}t}[\mathrm{P}] = -\sum_{\mathrm{X=V,A,Z}} k_{\mathrm{PX,F,eff}}[\mathrm{X}][\mathrm{P}] + \sum_{\mathrm{X=V,A,Z}} k_{\mathrm{PX,b}}[\mathrm{PX}] \tag{9}$$

$$\frac{\mathrm{d}}{\mathrm{d}t}[\mathrm{V}] = -k_{\mathrm{V\to A}}[\mathrm{VDEa}][\mathrm{V}] + k_{\mathrm{A\to V}}[\mathrm{A}] \tag{10}$$

$$\frac{\mathrm{d}}{\mathrm{d}t}[\mathrm{A}] = k_{\mathrm{V\to A}}[\mathrm{VDEa}][\mathrm{V}] - k_{\mathrm{A\to V}}[\mathrm{A}] - k_{\mathrm{A\to Z}}[\mathrm{VDEa}][\mathrm{A}] + k_{\mathrm{Z\to A}}[\mathrm{Z}] \tag{11}$$

$$\frac{\mathrm{d}}{\mathrm{d}t}[\mathrm{Z}] = k_{\mathrm{A\to Z}}[\mathrm{VDEa}][\mathrm{A}] - k_{\mathrm{Z\to A}}[\mathrm{Z}] \tag{12}$$

$$\frac{\mathrm{d}}{\mathrm{d}t}[\mathrm{VDEa}] = -\frac{\mathrm{d}}{\mathrm{d}t}[\mathrm{VDEi}] = k^{\mathrm{light/dark}}_{\mathrm{VDE,F,eff}}[\mathrm{VDEi}] - k^{\mathrm{light/dark}}_{\mathrm{VDE,b}}[\mathrm{VDEa}]. \tag{13}$$

The light/dark labeled rate constants take different values depending on the light conditions at a time $t$ in a given sequence of HL/D exposures, i.e.

$$k^{\mathrm{light/dark}} \equiv k^{\mathrm{light/dark}}(t) = \begin{cases} k^{\mathrm{light}}, \text{ if HL at time } t \\ k^{\mathrm{dark}}, \text{ if D at time } t. \end{cases} \tag{14}$$

There is some parametric redundancy in fitting the model to $\mathrm{NPQ}_\tau$ and HPLC data, specifically the model is independent of scaling $[\mathrm{VDE}]_{\mathrm{tot}} \to \gamma[\mathrm{VDE}]_{\mathrm{tot}}$, $k_{\mathrm{V\to A}} \to (1/\gamma)k_{\mathrm{V\to A}}$ and $k_{\mathrm{A\to Z}} \to (1/\gamma)k_{\mathrm{A\to Z}}$. As such we only work explicitly with the activity of VDE as a dynamical variable,

$$\alpha_{\mathrm{VDE}}(t) = \frac{[\mathrm{VDEa}]}{[\mathrm{VDEa}]^{\mathrm{light}}_{\mathrm{eq}}}, \tag{15}$$

where $[\mathrm{VDEa}]^{\mathrm{light}}_{\mathrm{eq}}$ is the equilibrium concentration of VDEa under light conditions, and we fit the maximum de-epoxidation rates, $k_{\mathrm{V\to A,\,max}} = k_{\mathrm{V\to A}}[\mathrm{VDEa}]^{\mathrm{light}}_{\mathrm{eq}}$ and $k_{\mathrm{A\to Z,\,max}} = k_{\mathrm{A\to Z}}[\mathrm{VDEa}]^{\mathrm{light}}_{\mathrm{eq}}$, and the response rate $k^{\mathrm{light/dark}}_{\mathrm{VDE}} = k^{\mathrm{light/dark}}_{\mathrm{VDE,f}} + k^{\mathrm{light/dark}}_{\mathrm{VDE,b}}$. Overall the equation for $\alpha_{\mathrm{VDE}}(t)$ is

$$\frac{\mathrm{d}}{\mathrm{d}t}\alpha_{\mathrm{VDE}}(t) = \alpha^{\mathrm{light/dark}}_{\mathrm{VDE,eq}} - k^{\mathrm{light/dark}}_{\mathrm{VDE}}(\alpha_{\mathrm{VDE}}(t) - \alpha^{\mathrm{light/dark}}_{\mathrm{VDE,eq}}) \tag{16}$$

where $\alpha^{\mathrm{light}}_{\mathrm{VDE,eq}} = 1$ and $\alpha^{\mathrm{dark}}_{\mathrm{VDE,eq}} = [\mathrm{VDEa}]^{\mathrm{dark}}_{\mathrm{eq}}/[\mathrm{VDEa}]^{\mathrm{light}}_{\mathrm{eq}}$.

As stated in the methods section, we work in reduced variables given by $[\widetilde{\mathrm{B}}] = \tau_F(0)k_{\mathrm{qE}}[\mathrm{B}]$, where $\tau_F(0)$ is the fluorescence lifetime at $t = 0$ and $k_{\mathrm{qE}}$ is the quenching rate associated with the QX species. With this the total $\mathrm{NPQ}_\tau$ is given by

$$\mathrm{NPQ}_\tau(t) = \frac{\tau_F(0) - \tau_F(t)}{\tau_F(t)} = \tau_F(0)(\tau_F(t)^{-1} - \tau_F(0)^{-1}) \tag{17}$$

$$= \tau_F(0)k_{\mathrm{qE}}\left(\Delta[\mathrm{QV}](t) + \Delta[\mathrm{QA}](t) + \Delta[\mathrm{QZ}](t) + \frac{k_{\mathrm{qZ}}}{k_{\mathrm{qE}}}\Delta[\mathrm{Z}](t)\right) \tag{18}$$

$$= \Delta[\widetilde{\mathrm{QV}}](t) + \Delta[\widetilde{\mathrm{QA}}](t) + \Delta[\widetilde{\mathrm{QZ}}](t) + \frac{k_{\mathrm{qZ}}}{k_{\mathrm{qE}}}\Delta[\widetilde{\mathrm{Z}}](t). \tag{19}$$

In order the model the VDE mutant $\mathrm{NPQ}_\tau$we account for the fact that the model predicts different fluorescence lifetimes for the WT and *vde* mutant,

$$\mathrm{NPQ}^{vde}_\tau(t) = \frac{1}{1 - [\widetilde{\mathrm{QV}}]^{vde}(0) + \sum_{\mathrm{X}}[\widetilde{\mathrm{QX}}]^{\mathrm{WT}}(0) + (k_{\mathrm{qZ}}/k_{\mathrm{qE}})[\widetilde{\mathrm{Z}}]^{\mathrm{WT}}(0)}\Delta[\widetilde{\mathrm{QV}}]^{vde}(t). \tag{20}$$

We find the correction factor to be almost exactly 1 (1.000006), which agrees with the very similar fluorescence lifetimes of the *vde* and WT species in the initial dark period of the experiments.

In fitting the model parameters we set the rate constants for the P+X binding and unbinding to be independent of the xanthophyll, and we also set the $k_{\mathrm{QX}}^{\mathrm{light/dark}} = k_{\mathrm{QX,f}}^{\mathrm{light/dark}} + k_{\mathrm{QX,b}}^{\mathrm{light/dark}}$ to be the same for all three xanthophylls. This reduces the number of free parameters and ensures that the only parameter controlling the efficacy of the xanthophylls as quencher is the equilibrium constant for the PX $\rightleftharpoons$ QX of a given xanthophyll. The parameters treated explicitly as free parameters are those given in Table I.

The kinetic equations for the model were solved using the "ode23s" solver in Matlab. Model parameters were fit to minimise the least squares difference between the model and experimental $\mathrm{NPQ}_\tau$

$$\mathcal{L} = \sum_{s,i} (\mathrm{NPQ}_\tau^{\mathrm{model}}(t_i; s) - \mathrm{NPQ}_\tau^{\mathrm{exp}}(t_i; s))^2 \tag{21}$$

where $s$ labels the sequences used in the fitting procedure: the 5 HL-9 D-5 HL, 5 HL-15 D-5 HL, 3 HL-1 D-1 HL-3 D-9 HL-3 D, 1 HL-2 D-7 HL-5 D-1 HL-2 D, 2 HL-2 D sequences. The parameters were fitted first using Matlab's "global search" function from an initial guess based on our previous model and HPLC data fits (described below). This was then refined using the "patternsearch" algorithm. Errors in the fitted parameters were estimated by bootstrapping the experimental data 1000 times and all reported errors are two standard deviations in the mean of the bootstrapped parameter distributions.

## II. REDUCED MODEL FOR HPLC DATA

In order to obtain first estimates of the xanthophyll epoxidation/de-epoxidation rates, we fitted the HPLC data directly to a reduced version of the full. We obtain this reduced model by assuming the binding/unbinding time-scales and PX $\rightleftharpoons$ QX time-scales are fast compared to the xanthophyll interconversion. With this we can invoke a quasi-equilibrium approximation for the P,X,PX and QX species.

$$[\mathrm{QX}] \approx K_{\mathrm{QX}}^{\mathrm{light/dark}}[\mathrm{PX}] \tag{22}$$

$$[\mathrm{PX}] \approx K_{\mathrm{PX}}[\mathrm{P}][\mathrm{X}]. \tag{23}$$

With this we find the pool X concentration is

$$[\mathrm{X}] \approx \frac{1}{1 + K_{\mathrm{PX,eff}}[\mathrm{P}]}[\mathrm{X}]_{\mathrm{tot}} \tag{24}$$

$$K_{\mathrm{PX,eff}} = (1 + K_{\mathrm{QX}}^{\mathrm{light/dark}})K_{\mathrm{PX}}, \tag{25}$$

and therefore the rate of xanthophyll interconversion is given by

$$\frac{\mathrm{d}}{\mathrm{d}t}[\mathrm{V}]_{\mathrm{tot}} = -\alpha_{\mathrm{VDE}}(t)\frac{k_{\mathrm{V}\to\mathrm{A,max}}}{1 + K_{\mathrm{PV,eff}}[\mathrm{P}]}[\mathrm{V}]_{\mathrm{tot}} + \frac{k_{\mathrm{A}\to\mathrm{V}}}{1 + K_{\mathrm{PA,eff}}[\mathrm{P}]}[\mathrm{A}]_{\mathrm{tot}} \tag{26}$$

$$\frac{\mathrm{d}}{\mathrm{d}t}[\mathrm{A}]_{\mathrm{tot}} = \alpha_{\mathrm{VDE}}(t)\frac{k_{\mathrm{V}\to\mathrm{A,max}}}{1 + K_{\mathrm{PV,eff}}[\mathrm{P}]}[\mathrm{V}]_{\mathrm{tot}} - \frac{k_{\mathrm{A}\to\mathrm{V}}}{1 + K_{\mathrm{PA,eff}}[\mathrm{P}]}[\mathrm{A}]_{\mathrm{tot}} - \alpha_{\mathrm{VDE}}(t)\frac{k_{\mathrm{A}\to\mathrm{Z,max}}}{1 + K_{\mathrm{PA,eff}}[\mathrm{P}]}[\mathrm{A}]_{\mathrm{tot}} + \frac{k_{\mathrm{Z}\to\mathrm{A}}}{1 + K_{\mathrm{PZ,eff}}[\mathrm{P}]}[\mathrm{Z}]_{\mathrm{tot}} \tag{27}$$

$$\frac{\mathrm{d}}{\mathrm{d}t}[\mathrm{Z}]_{\mathrm{tot}} = \alpha_{\mathrm{VDE}}(t)\frac{k_{\mathrm{A}\to\mathrm{Z,max}}}{1 + K_{\mathrm{PA,eff}}[\mathrm{P}]}[\mathrm{A}]_{\mathrm{tot}} - \frac{k_{\mathrm{Z}\to\mathrm{A}}}{1 + K_{\mathrm{PZ,eff}}[\mathrm{P}]}[\mathrm{Z}]_{\mathrm{tot}}. \tag{28}$$

Because the "pool" xanthophylls are in excess [P] is very small so it can be treated as being in steady state, so we assume that $K_{\mathrm{PX}}[\mathrm{P}]$ can be treated as constant. This means estimates of the xanthophyll interconversion rates can be obtained using a first order kinetic model with light-phase dependent rate constants, and the VDE activation as treated in the full model.

## III. MODEL PARAMETERS

The final set of fitted model parameters are given in Table I, obtained from least squares fitting of a subset of the $\mathrm{NPQ}_\tau$data with xanthophyll interconversion rate constants constrained to be within 50% of values obtained from the reduced model fitting. The reduced model fitting produced rate constants of $k_{\mathrm{V}\to\mathrm{A,max}} = 0.1307$ min$^{-1}$, $k_{\mathrm{A}\to\mathrm{Z,max}} = 0.0918$ min$^{-1}$, $k_{\mathrm{Z}\to\mathrm{A,max}} = 0.1245$ min$^{-1}$, $k_{\mathrm{A}\to\mathrm{V,max}} = 0.0458$ min$^{-1}$, $\alpha_{\mathrm{VDE,eq}}^{\mathrm{dark}} = 0.0013$, $k_{\mathrm{VDE}}^{\mathrm{light}} = 1.285$ min$^{-1}$, and $k_{\mathrm{VDE}}^{\mathrm{dark}} = 1.019$ min$^{-1}$.

In comparing the model HPLC data to the experimental HPLC data, we found a scaling constant of 0.98 mmol / mol Chl between the reduced units of the model and the concentration relative the Chl by least squares fitting the full model HPLC predictions to the experimental values. From this we can estimate the total concentration of LHCX1 (P in the model) to be about 3.5 mmol / mol Chl.

| Parameter | Value | Lower bound (95% CI) | Upper bound (95% CI) |
|---|---|---|---|
| $k_{A\rightarrow Z,\,max}$ | 0.1361 | 0.0935 | 0.1951 |
| $k_{V\rightarrow A,\,max}$ | 0.0918 | 0.0688 | 0.1181 |
| $k_{Z\rightarrow A}$ | 0.0854 | 0.0832 | 0.1414 |
| $k_{A\rightarrow V}$ | 0.0509 | 0.0307 | 0.0685 |
| $k^{light}_{VDE}$ | 1.2846 | 1.1954 | 1.2962 |
| $k^{dark}_{VDE}$ | 1.0193 | 0.5732 | 76.4352 |
| $\alpha^{dark}_{VDE,\,eq}$ | 0.0010 | 0.0009 | 0.0019 |
| $k_{PV,\,b}$ | 3.4187 | 1.6648 | 10.8108 |
| $k_{PA,\,b}$ | 3.4187 | 1.6648 | 10.8108 |
| $k_{PZ,\,b}$ | 3.4187 | 1.6648 | 10.8108 |
| $k^{light}_{QZ}$ | 2.0744 | 1.9304 | 2.3957 |
| $k^{dark}_{QZ}$ | 4.6913 | 4.3656 | 8.3424 |
| $K_{PV}$ | 0.2400 | 0.2079 | 1623.4242 |
| $K_{PA}$ | 0.2400 | 0.2079 | 1623.4242 |
| $K_{PZ}$ | 0.2400 | 0.2079 | 1623.4242 |
| $K^{light}_{QZ}$ | 10.9158 | 3.4519 | 14.5586 |
| $K^{dark}_{QZ}$ | 0 | – | – |
| $[\widetilde{V}]_0$ | 67.9332 | 67.6284 | 69.1663 |
| $[\widetilde{P}]_{tot}$ | 3.5324 | 3.5245 | 3.5326 |
| $K^{light}_{QA}$ | 0.3872 | 0.0353 | 0.5163 |
| $K^{dark}_{QA}$ | 0 | – | – |
| $k^{light}_{QA}$ | 2.0744 | 1.9304 | 2.3957 |
| $k^{dark}_{QA}$ | 4.6913 | 4.3656 | 8.3424 |
| $K^{light}_{QV}$ | 0.1173 | 0.0879 | 0.1175 |
| $K^{dark}_{QV}$ | 0 | – | – |
| $k^{light}_{QV}$ | 2.0744 | 1.9304 | 2.3957 |
| $k^{dark}_{QV}$ | 4.6913 | 4.3656 | 8.3424 |
| $k_{qZ}/k_{qE}$ | 0.0259 | 0.0234 | 0.0343 |

TABLE I. Best fit parameters obtained for the full model. Confidence intervals obtained by bootstrapping experimental NPQ runs and estimating 95% confidence intervals from the approximate parameter distribution. All parameters are given in reduced units of the model, therefore all rate constants are in $\min^{-1}$ and all other parameters are unitless.

## IV. MECHANISM OF QZ

In our model we treat the qZ quenching process as an additional first order quenching process just proportional to the concentration of "pool" Zeaxanthin. We can arrive at this model using a simple model similar to our LHCX1 based quenching model. We consider adding a second protein or complex to our model denoted P′, which binds xanthophylls to form complexes PX′ = PV′, PA′, PZ′. We assume the quenching of chlorophyll excitations is proportional to the concentration of PZ′, such that the change in fluroescence decay rate is $\Delta k_{F,qZ} = k_{Q,PZ'}[PZ']$. Assuming that P' binding X can be treated witht he pre-equilibrium/quasi-equilibrium approximation, we find that

$$[PX'] = \frac{K_{PX'[P]}[X]_{pool}}{K_{PX'}[P]+1}. \tag{29}$$

where $[X]_{pool}$ is the xanthophyll concentration in the pool including that bound to P', and $K_{PX}$ is the equilibrium constant for P' binding X. Assuming that P' is in a steady state, where $\frac{d}{dt}[P](t) \approx 0$, and thus $[P](t) \approx [P]_0$, the change in quenching rate due to qZ is simply proportional to $[Z]_{pool}$, as is assumed in the model.

## V. ESTIMATING QUENCHING RATES

We can construct a simple model for excitation quenching as follows. We assume that the excited chlorophylls, Chl*, can exist either on an active quenching complex, QX, which we label $\mathrm{Chl}^*_\mathrm{Q}$, or on the other light-harvesting complexes, which we label $\mathrm{Chl}^*_\mathrm{Pool}$. We treat the populations of Chl* in these two environment with a simple first order kinetic model, with a diffusion rate onto QX of $\eta_\mathrm{Q} k_\mathrm{D}$ and a diffusion rate off the QX site given by $k_\mathrm{D}$. $\eta_\mathrm{Q}$ is the ratio of the number of Chl on QX to the number of Chl in the whole system, which we estimate to be approximately the ratio of QX to the all of the light harvesting proteins. We further assume that the rate of decay of the Chl* down to its ground state is dependent on the site, occurring at a rate $k_{\mathrm{F,eff},0}$ (Chl* decay is dominated by non-radiative decay, but this rate constant should be understood as including a small radiative contribution) in the pool and at a rate $k_{\mathrm{F,eff,Q}}$ on the quenching sites. Putting these ingredient together we arrive at the following kinetic equations for $\mathrm{Chl}^*_\mathrm{Q}$ and $\mathrm{Chl}^*_\mathrm{Pool}$

$$\frac{\mathrm{d}}{\mathrm{d}t}[\mathrm{Chl}^*_\mathrm{Q}] = -(k_{\mathrm{F,eff,Q}} + k_\mathrm{D})[\mathrm{Chl}^*_\mathrm{Q}] + \eta_\mathrm{Q} k_\mathrm{D}[\mathrm{Chl}^*_\mathrm{Pool}] \tag{30}$$

$$\frac{\mathrm{d}}{\mathrm{d}t}[\mathrm{Chl}^*_\mathrm{Pool}] = -(k_{\mathrm{F,eff},0} + \eta_\mathrm{Q} k_\mathrm{D})[\mathrm{Chl}^*_\mathrm{Pool}] + k_\mathrm{D}[\mathrm{Chl}^*_\mathrm{Q}]. \tag{31}$$

Applying the steady state approximation to $[\mathrm{Chl}^*_\mathrm{Q}]$, we obtain the following equation for the decay of the pool Chl*,

$$\frac{\mathrm{d}}{\mathrm{d}t}[\mathrm{Chl}^*_\mathrm{Pool}] \approx -\left(k_{\mathrm{F,eff},0} + \eta_\mathrm{Q} k_\mathrm{D}\frac{k_{\mathrm{F,eff,Q}}}{k_{\mathrm{F,eff,Q}} + k_\mathrm{D}}\right)[\mathrm{Chl}^*_\mathrm{Pool}], \tag{32}$$

from which we obtain the fluorescence lifetime as

$$\frac{1}{\tau_{\mathrm{F,eff}}} = k_{\mathrm{F,eff},0} + \eta_\mathrm{Q} k_\mathrm{D}\frac{k_{\mathrm{F,eff,Q}}}{k_{\mathrm{F,eff,Q}} + k_\mathrm{D}}, \tag{33}$$

recalling that $\eta_\mathrm{Q} \propto \sum_\mathrm{X}[\mathrm{QX}]$, the expression we find is consistent with the assumptions of our NPQ model (excluding qZ). Assuming $\eta_\mathrm{Q} \approx 0$ before light exposure, we find the $\mathrm{NPQ}_\tau$ as

$$\mathrm{NPQ}_\tau = \eta_\mathrm{Q}\frac{k_\mathrm{D}}{k_{\mathrm{F,eff},0}}\frac{k_{\mathrm{F,eff,Q}}}{k_{\mathrm{F,eff,Q}} + k_\mathrm{D}}. \tag{34}$$

If we assume excitation energy diffusion is very fast between proteins compared to the other time-scales in the model, we find that the $\mathrm{NPQ}_\tau$] is given approximately by

$$\mathrm{NPQ}_\tau = \eta_\mathrm{Q}\frac{k_{\mathrm{F,eff,Q}}}{k_{\mathrm{F,eff},0}}. \tag{35}$$

From the time-correlated photon counting experiments used to obtain the $\mathrm{NPQ}_\tau$ we know $k_{\mathrm{F,eff},0} \approx 1\ \mathrm{ns}^{-1}$. The maximum $\mathrm{NPQ}_\tau$ within our model is limited by the total concentration of P (in reduced units), $[\widetilde{\mathrm{P}}]_\mathrm{tot} \sim 3.5$. From the HPLC experiments we have deduced that P is present at a concentration of around 3.5 mmol / mol Chl. Assuming ∼ 10 Chl per light-harvesting protein, this means about 1 in 30 proteins in the chloroplast would be P, which puts an upper bound on $\eta_\mathrm{Q}$ of $\sim 1/30$. From this we can estimate a lower bound on $k_{\mathrm{F,eff,Q}}$ to be $k_{\mathrm{F,eff,Q}} \sim 100\ \mathrm{ns}^{-1}$, i.e. the lifetime of Chl* on the quencher must be ∼ 10 ps. If we instead use $(1/7.7)\ \mathrm{ps}^{-1}$ as an estimate for $k_{\mathrm{F,eff,Q}}$, as obtained in Ref. 1, we deduce that roughly 1 in 43 light-harvesting proteins in the chloroplast are P. Given the large simplifications and the uncertainty in the abundance of P deduced from HPLC data and the model (due to the large uncertainty in the conversion factor from model concentration to abundance in the thylakoid membrane), we consider these estimates of the proportion of P and the quenching lifetimes as being in excellent agreement.

## VI. RAW HPLC DATA

In Fig. 1 we show the raw HPLC data for each of the HL/D sequences shown in the main text. A certain fraction of each xanthophyll does not change over the course of the experiment. Since our model only includes xanthophylls that are free to bind/unbind from proteins on the time-scale of our experiments, we only examine the changes in xanthophyll concentration, and use these changes in fitting the model.

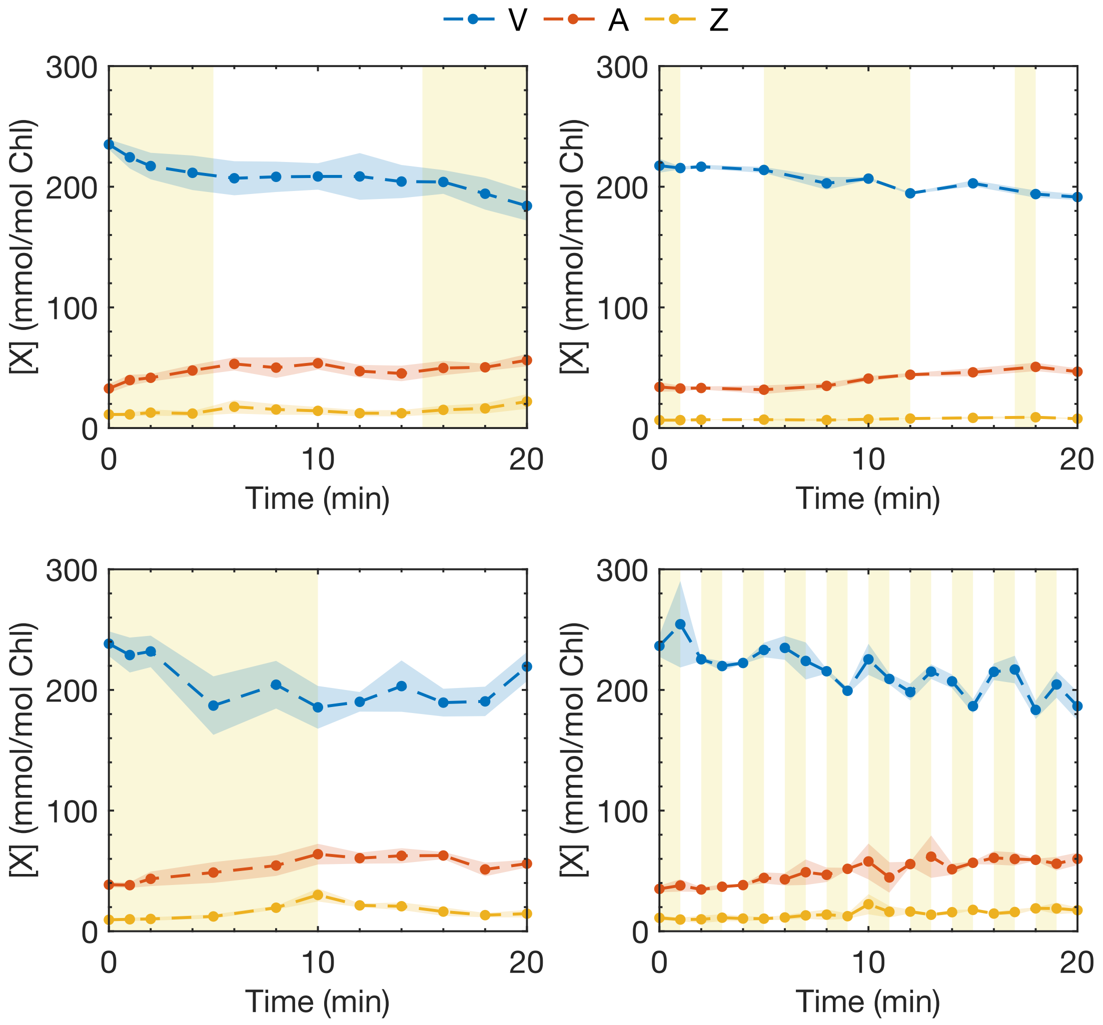


FIG. 1. Raw HPLC data for concentrations of each xanthophyll normalized by the total Chl concentration for four HL/D sequences: 5 HL- 10 D- 5 HL (top left), 1 HL- 4 D- 7 HL- 5 D- 1 HL- 2 D (top right), 10 HL- 10 D (bottom left), 1 HL-1 D (bottom right).

## VII. NPQ RECOVERY IN 5 HL-$T$ D-5 HL SEQUENCES

In Fig. 2 we show window averaged $\mathrm{NPQ}_\tau$ in the second light phase for the 5 HL-$T$ D-5 HL sequences, normalised by the $\mathrm{NPQ}_\tau$ value at $t = 5$ min. This window averaging is defined as

$$\overline{\mathrm{NPQ}_\tau} = \frac{1}{t_f - t_i} \int_{t_i}^{t_f} \mathrm{NPQ}_\tau(t)\,\mathrm{d}t\,. \tag{36}$$

The experimental window averaging is estimated using the trapezoidal rule. Fitting the averaged normalised $\mathrm{NPQ}_\tau$ in the first minute to an exponential decay as a function of $T$, i.e. $\overline{\mathrm{NPQ}_\tau} = \overline{\mathrm{NPQ}_{\tau,0}} e^{-k_{\mathrm{mem}} T}$, we obtain an effective recovery rate constant of $k_{\mathrm{mem}} = 0.0464$ (lower CI (95%): 0.0064, upper CI (95%): 0.0861) $\mathrm{min}^{-1}$, which matches the model $k_{\mathrm{A} \longrightarrow \mathrm{V}}$ rate constant of 0.0509 $\mathrm{min}^{-1}$.

## REFERENCES

[1]S. Park, C. J. Steen, D. Lyska, A. L. Fischer, B. Endelman, M. Iwai, K. K. Niyogi, and G. R. Fleming, "Chlorophyll–carotenoid excitation energy transfer and charge transfer in Nannochloropsis oceanica for the regulation of photosynthesis," Proceedings of the National Academy of Sciences of the United States of America **116**, 3385—3390 (2019).

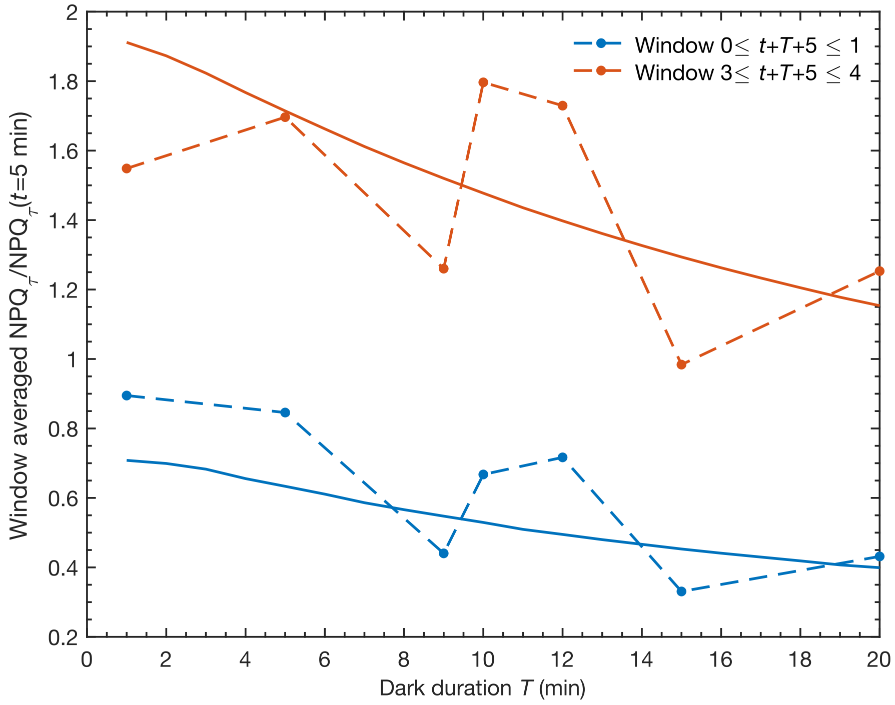


FIG. 2. Window averaged NPQ$_\tau$ in the second light phase for the 5 HL-$T$ D-5 HL sequences, normalised by the NPQ$_\tau$ value at $t$ = 5 min, for the first minute (blue) and fourth minute (red) of the second light phase, from the experiment (dashed lines and circles) and model (solid line). Error bars correspond to two standard errors in the mean. The data collection/number of replicates is described in the methods section of the main text.
